# Supplementary material for: A genome-wide arrayed CRISPR screen identifies PLSCR1 as an intrinsic barrier to SARS-CoV-2 entry that recent virus variants have evolved to resist
Source: PLoS Biol. 2024 Sep 24;22(9):e3002767. doi: 10.1371/journal.pbio.3002767 (PMC11486371; doi:10.1371/journal.pbio.3002767)

Supplementary figure 1B

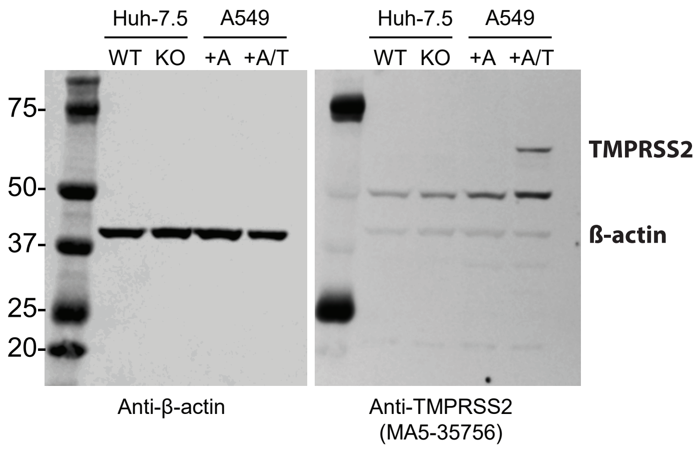

Supplementary figure 5A

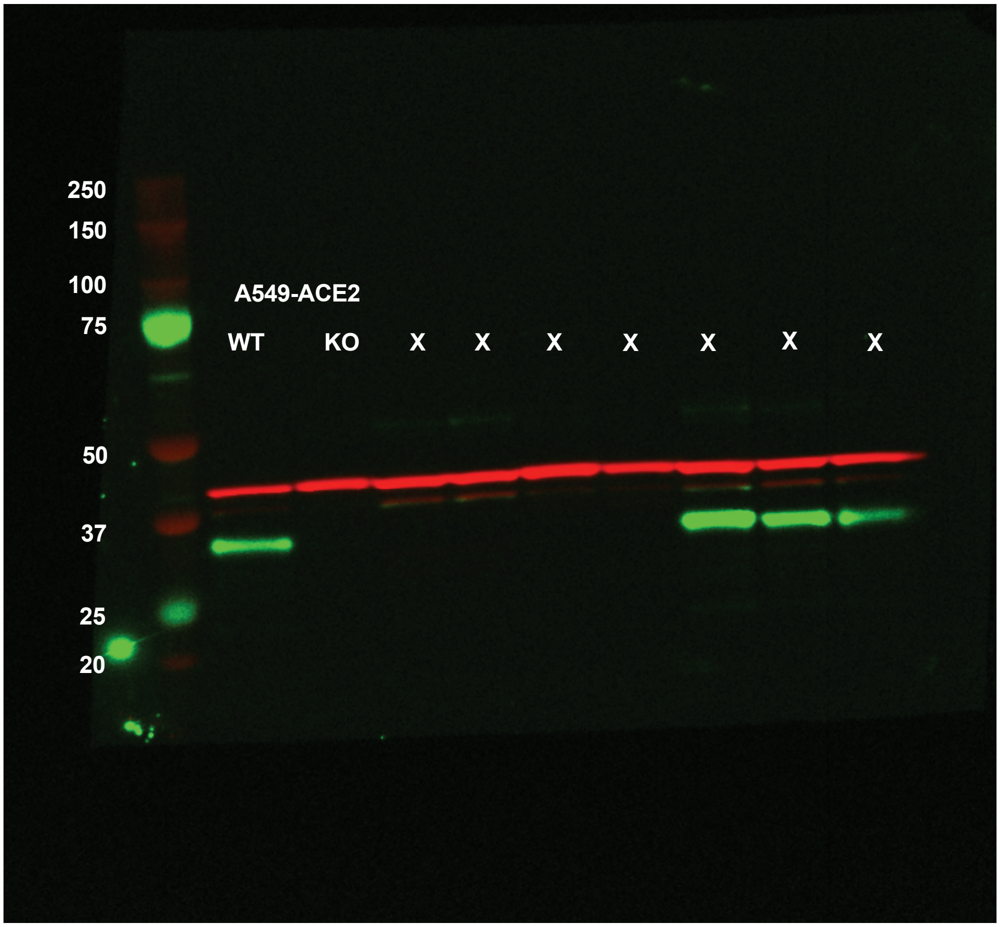

β-actin

PLSCR1

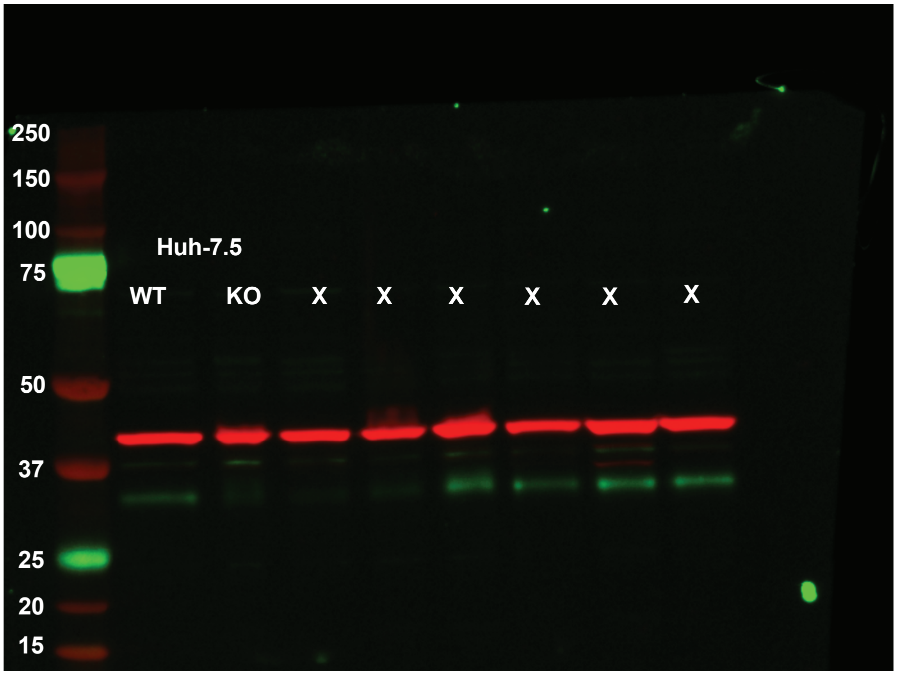

β-actin

PLSCR1

Supplementary figure 5C

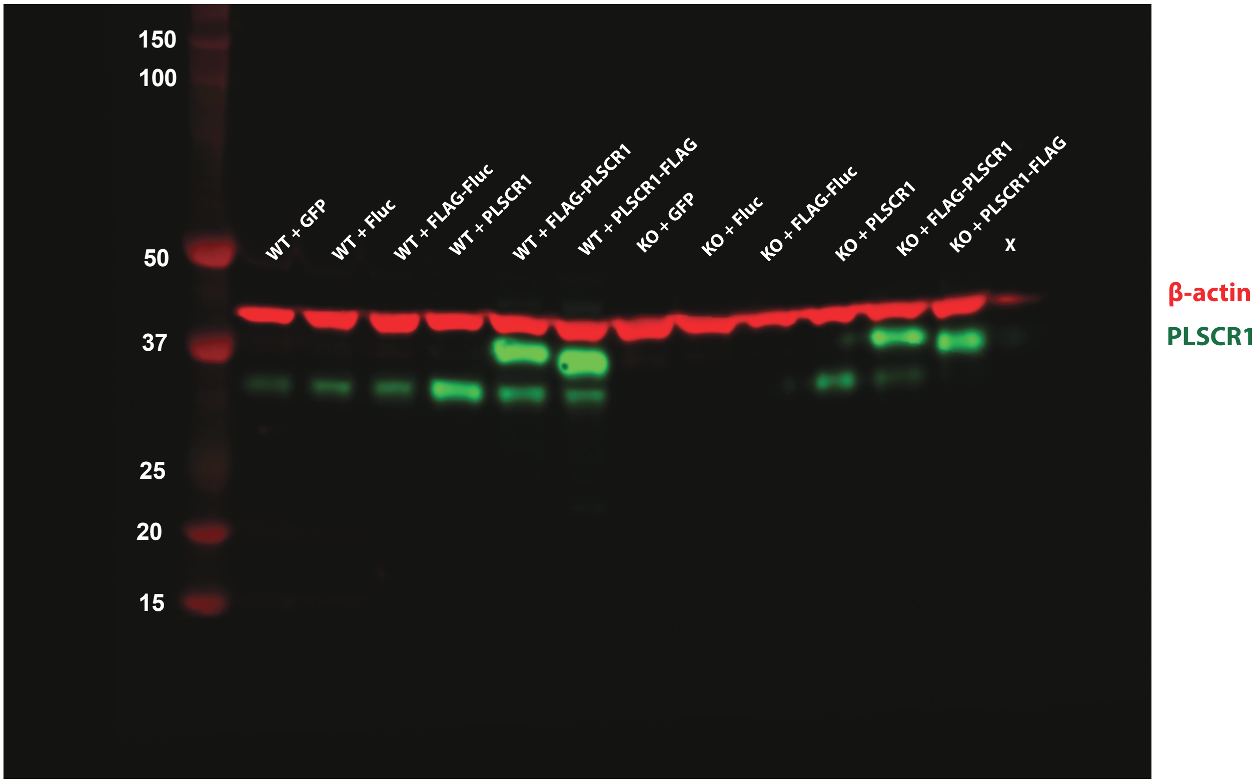

Supplementary figure 7A

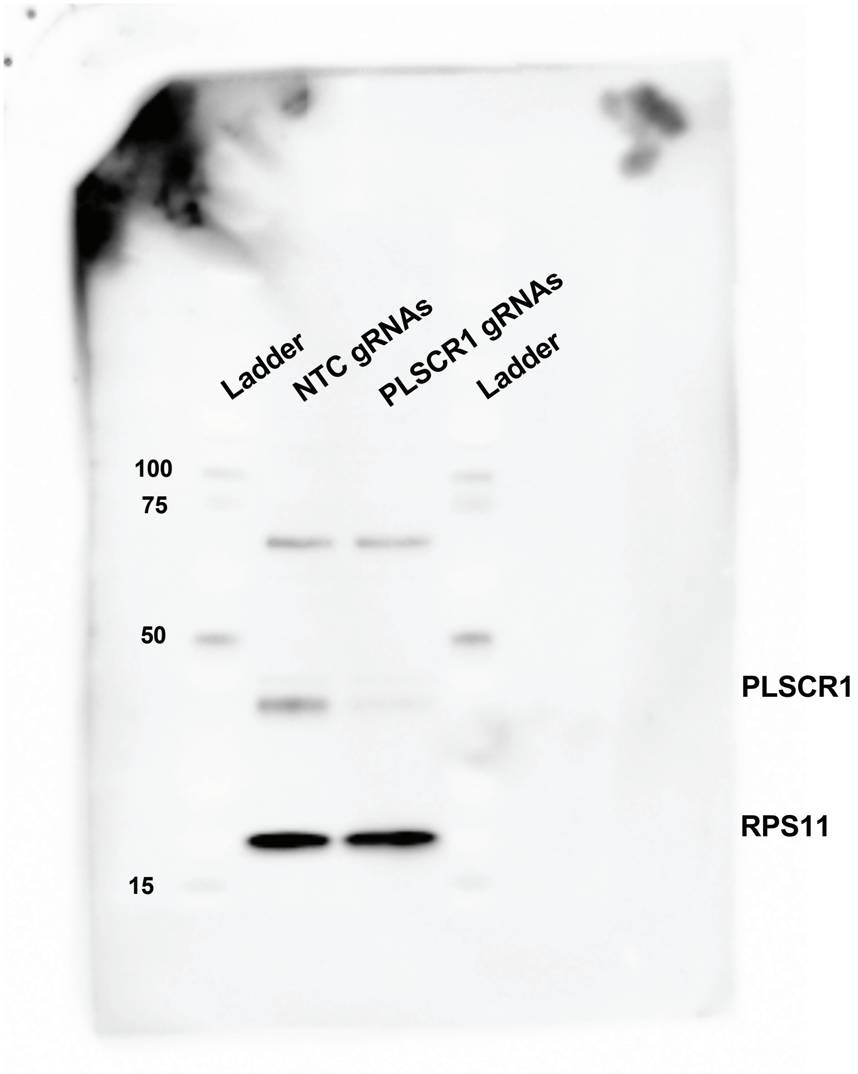

Supplementary figure 11A

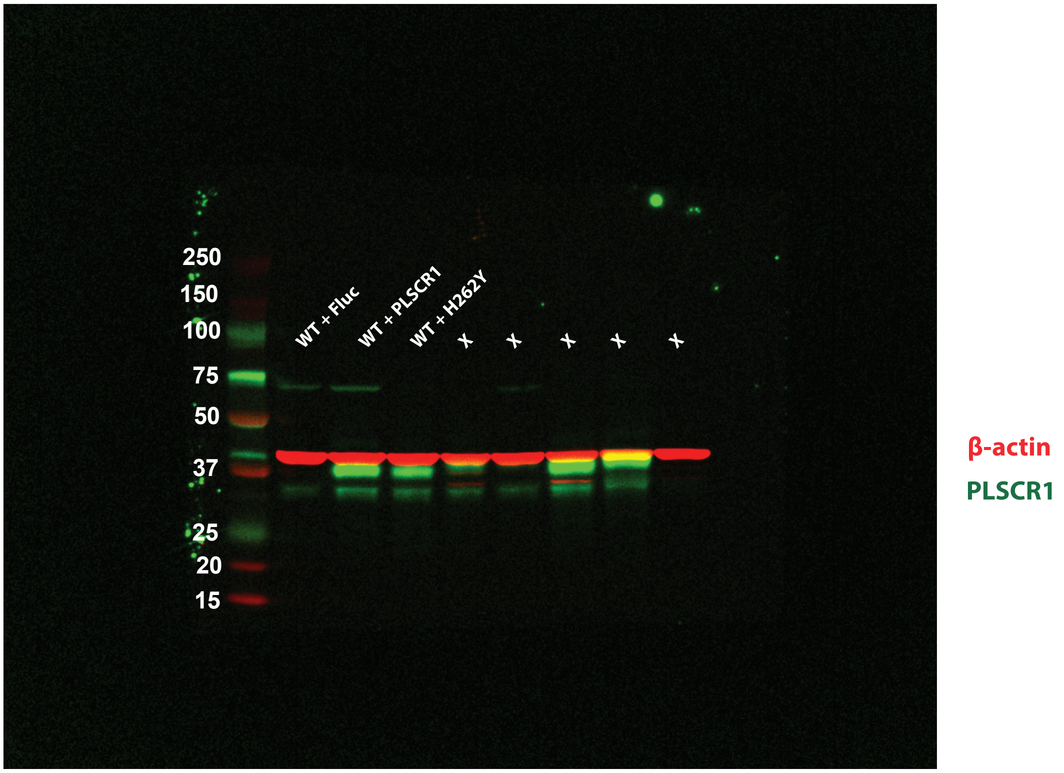

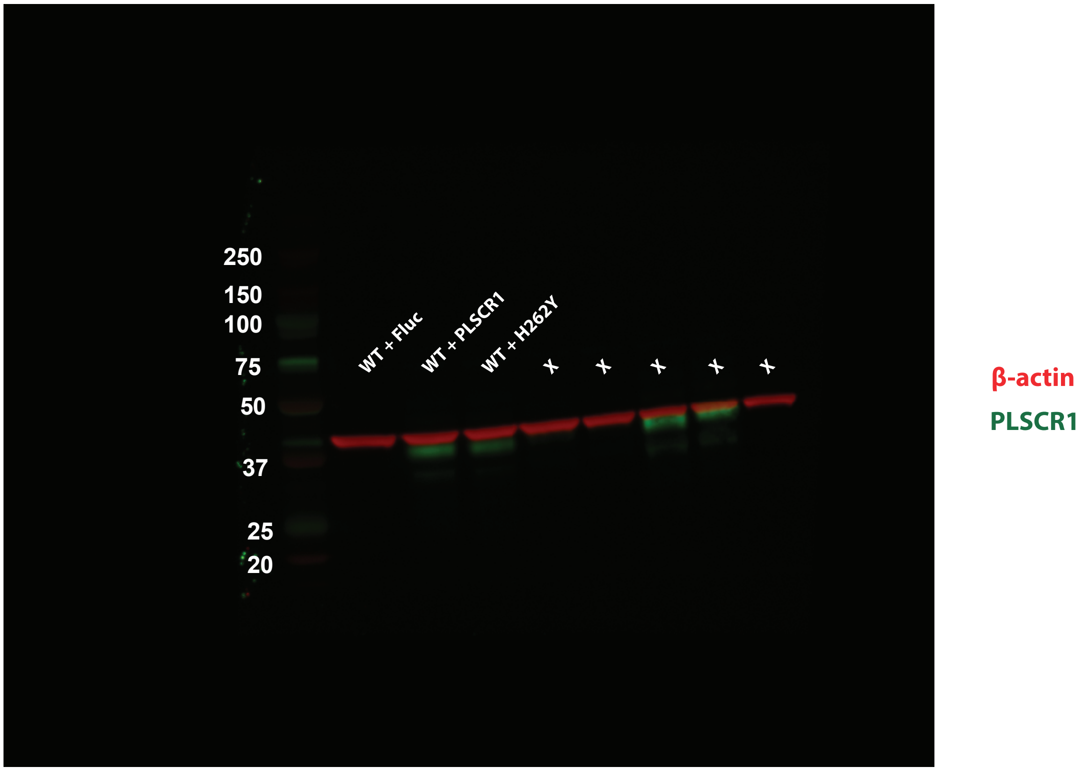

Supplement: S1 Supporting Information — (ZIP) [file pbio.3002767.s035.zip › Supporting Information 1 Raw images/Supp Information 1 Western Blots Raw Images.pdf]
