# Supplementary figures and images for: A genome-wide arrayed CRISPR screen identifies PLSCR1 as an intrinsic barrier to SARS-CoV-2 entry that recent virus variants have evolved to resist
Source: PLoS Biol. 2024 Sep 24;22(9):e3002767. doi: 10.1371/journal.pbio.3002767 (PMC11486371; doi:10.1371/journal.pbio.3002767)

Le Pen et al. Supporting Information 2, Supp Fig 10 Gating Strategy

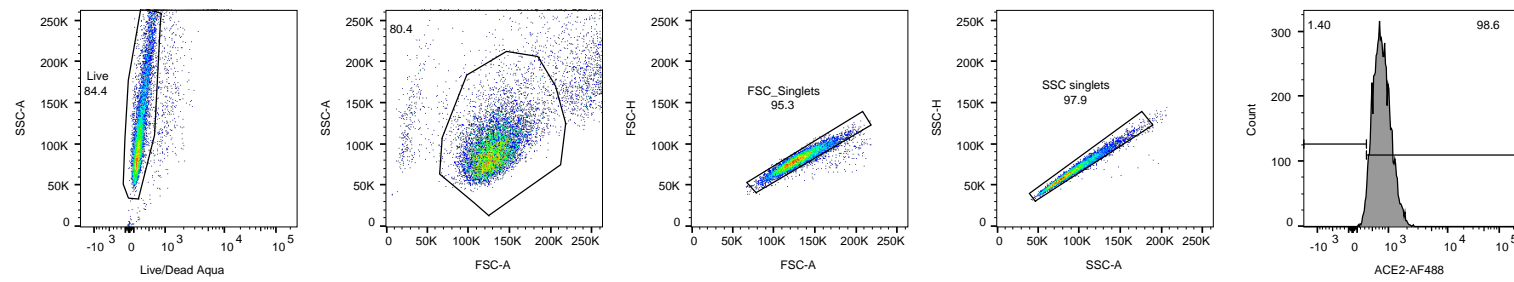

Supplement: S2 Supporting Information — (ZIP) [file pbio.3002767.s036.zip › Supporting Information 2 Flow cytometry gating strategy/Supp Information 2 Flow Gating Strategy.pdf]
