# Supplementary material for: A genome-wide arrayed CRISPR screen identifies PLSCR1 as an intrinsic barrier to SARS-CoV-2 entry that recent virus variants have evolved to resist
Source: PLoS Biol. 2024 Sep 24;22(9):e3002767. doi: 10.1371/journal.pbio.3002767 (PMC11486371; doi:10.1371/journal.pbio.3002767)
Supplement: Attachment — Submitted filename: Le Pen et al. Response to Reviewers version 14.pdf [file pbio.3002767.s038.pdf]

Dear Editor,

We appreciate the reviewers' thorough evaluation of our manuscript, "A Genome-Wide Arrayed CRISPR Screen Reveals PLSCR1 as an Intrinsic Barrier to SARS-CoV-2 Entry," and their excellent feedback. Below, we address each comment point by point.

Reviewer 1

General Comments

Comment: This manuscript describes efforts to identify new modulators of SARS-CoV-2 infection using an arrayed CRISPR KO screening approach. The screen is done using Huh-7.5 cells, which are probably not representative of a primary SARS-CoV-2 target cell, but they are naturally permissive. The investigators treat cells with IFN and then evaluate infection with authentic SARS-CoV-2. The investigators focus on one gene, PLSCR1 as an innate cellular defense component, and a similar observation was recently reported in Nature. The study is well designed and the data of reasonably high quality. Below are comments that are intended to improve the quality of the manuscript.

Response: We thank the reviewer for their positive comments on our study design and data quality. Below, we address the specific points raised to further improve the manuscript.

Specific Comments

1. Figure 1:

Comment: Figure 1 might be better adapted and used instead as a visual abstract, or placed in supplemental.

Response: We have moved Figure 1 (in the previous version) to the supplemental section, Supplemental Figure 1 (in the updated version).

2. Essential Genes:

Comment: The number of "essential genes (16790 - 16178 = 612) seems low compared to pooled CRISPR screen data. Is this because of the shorter culture time from KO to assay?

Response: The shorter culture time in the arrayed screen compared to pooled screens indeed contributes to the lower number of essential genes detected. It can take more than 24 doublings for some essential genes to be fully depleted [1]. We have clarified this in the revised manuscript (see Introduction section).:

Here, we conducted a human whole-genome arrayed CRISPR KO screen to identify genes that influence SARS-CoV-2 infection in cells with or without pretreatment with a low dose of IFN. The arrayed approach, though logistically challenging, has advantages over the pooled format in capturing both proviral and antiviral genes, genes affecting virus egress, and those coding for secreted products that exert their impact on neighboring cells. It reliably captures genotype-phenotype correlations while also unveiling the effects of single gene perturbation on cell growth and death [20]. Additionally, the shorter culture time and lack of competition among cells with different gene KO in the arrayed screen allow the inclusion of genes that would be depleted and deemed essential in a pooled format [21]. The arrayed format thus enables the identification of crucial cellular functions that may be co-opted by the virus or are vital for the cell's defense against infection.

---

3. TMPRSS2-Dependent Fusion Pathway:

Comment: As I understand, the TMPRSS2-dependent fusion pathway is the primary entry pathway used *in vivo*. So does screening for modifiers of the endocytic pathway teach use important information about host pathogen interactions? There would be some overlap, but is this data primarily of interest to *in vitro* infections only? This could be discussed to help the reader understand where this data fits in the bigger picture.

Response: We now discuss this point (see Discussion section).:

The original SARS-CoV-2 strain can enter cells through both TMPRSS2-dependent fusion near the cell surface and clathrin-mediated endocytosis, with a preference for the former in vivo [38]. ISGs that primarily restrict SARS-CoV-2 endocytosis, such as NCOA7, which perturbs lysosome acidification [161, 162], and PLSCR1, may play a role in constraining the original strain to TMPRSS2-dependent entry. Interestingly, the more recent Omicron variant shows both increased evasion from PLSCR1 restriction (Fig 6) and an acquired preference for TMPRSS2-independent entry. This occurs either via endocytosis or TMPRSS2-independent fusion near the cell surface, utilizing metalloproteases to cleave the spike protein [160, 163, 164]. We postulate that Omicron evolved its entry pathway to evade

PLSCR1-mediated restriction while shifting its cell tropism to the upper airways and away from TMPRSS2-expressing cells [163, 165-169].

---

4. Total # of Resistant and Sensitive Hits:

Comment: The authors should include the total # of resistant and sensitive hits identified in the screen in the text with a reference to table s7.

Response: We have added the total number of hits to the text (see Results section).:

---

*We classified 448 genes with a SARS-CoV-2 infection z-score ≥ 2 as antiviral hits and 507 genes with a SARS-CoV-2 infection z-score ≤ -2 as proviral hits (**Supp Table 7**).*

Comment: It would probably be easier to understand this table if z score was included here as well.

Response: We have added the z-score to **Supp Table 7**.

Comment: For comparison between these data and previous CRISPR screens, can the authors include some information on the % of overlap with each KO? Is there significant enrichment between hit lists compared to random data? Or is each screen showing a different and complementary perspective on host virus interactions? Might need to adjust the z score for this analysis since this arrayed screen has many more hits than most of the pooled screens. It would be of general interest to know how well the various screens complement or reinforce each other. GSEA can often give a different perspective than just straight pathway analysis, can the authors include standard pathway analysis for the ~1000 hits at least in supplemental? This kind of analysis can help the reader understand the core mechanisms identified that themselves directly impact infection.

Response: We appreciate this suggestion. We have now added a general comparison between the unbiased pooled screens and our arrayed screen (see Results section):

The overlap of gene hits influencing SARS-CoV-2 between our arrayed screen and fifteen published whole-genome pooled screens [19, 31, 64-67, 69-71, 74, 75, 77-80] was higher than expected by chance (Supp. Fig 4). This finding indicates that genetic screens conducted with different methods

> *(e.g., CRISPR activation or CRISPR KO, pooled or arrayed format) and in various cellular contexts (e.g., Huh-7.5, A549-ACE2, Calu-3, with or without IFN pretreatment) exhibit both specificity and significant overlap. A pathway analysis of the hits from our arrayed screen, alongside hits from pooled screens, is available in Supp. Table 11.*

The new **Supp. Table 11** also addresses the reviewer's suggestion of a standard pathway analysis for the ~1000 hits in the supplemental material.

Additionally, we have added a comparison of the overall number of genes classified as hits in different SARS-CoV-2 unbiased screen publications (**Supp. Fig 4**). Although our arrayed screen is among the published screens with the highest number of genes classified as hits, this is reasonable given that we tested two conditions (with and without IFN pretreatment) and that the arrayed format allowed us to screen essential cellular functions such as translation. We do not think that the z-score needs to be adjusted. Instead, we provide all the necessary data tables and summary tables for interpreting our screen data independently of this threshold and without the need for extra bioinformatics analyses.

5. Figure 2:

Comment: Please add a few more sentences discussing the changes observed in 2A-C to better set up the study design.

Response: We have added a few more sentences to better set up the study design (see Results section):

> *Huh-7.5 cells are defective in virus sensing and do not commonly produce IFN during infection [35]. We confirmed that Huh-7.5 cells do not induce ISG expression during SARS-CoV-2 infection (Fig 1A, Supp. Tables 1 and 2). This is likely due to a defect upstream of IFN production, as these cells did induce ISG expression when treated with recombinant IFN (Fig 1B, Supp. Tables 3 and 4). Thus, Huh-7.5 cells are a convenient model for studying controlled IFN responses during viral infection. Furthermore, IFN treatment restricted SARS-CoV-2 (Fig 1C), indicating that some ISGs are effective in limiting SARS-CoV-2 infection in Huh-7.5 cells. This allows us to study the functional landscape of SARS-CoV-2 restriction, examining both intrinsic*

factors and those induced in response to IFN signaling. Similar to A549-ACE2 cells [11, 36], Huh-7.5 cells do not express transmembrane serine protease 2 (TMPRSS2) (Supp Fig 1B). As a result, SARS-CoV-2 entry is restricted to the endocytic route [37, 38].

Comment: The authors should present the positive and negative control values separately so the reader can see the overall reproducibility of this approve when targeting genes with known effects.

Response: We now present the control values separately in **Fig 1E** and in the text (see Results section):

We included control gRNAs with known proviral and antiviral effects in our screening plates. As expected, the SARS-CoV-2 receptor angiotensin-converting enzyme 2 (ACE2) [9] behaved as a proviral gene with and without IFN pretreatment. The interferon-alpha/beta receptor alpha chain 1 (IFNAR1) [36-38] was essential for IFN treatment to restrict SARS-CoV-2. The ISG15 ubiquitin-like modifier (ISG15) has been previously described as a negative regulator of the IFN response. ISG15 stabilizes ubiquitin-specific peptidase 18 (USP18) [39-41], which in turn binds to the interferon-alpha/beta receptor alpha chain 2 (IFNAR2) and blocks signal transduction [42, 43]. Accordingly, ISG15 was proviral in IFN-treated cells (Fig 1E).

Comment: 2E please darken the lines that set up the various response quadrants. It was slightly difficult to access these data in 2E, for some people at least its easier to just describe hits as sensitive or resistant as phenotype, vs describing sensitive as "antiviral", and resistant as "proviral", then convert the phenotype data from 2E (resistant vs sensitive) to function of the gene (proviral or antiviral) in 2F. This is not essential. Regardless, some effort to make this analysis more intuitive to the reader can increase the overall ability for more naïve readers to access the work.

Response: **Figure 2E** (in the previous version) was discussed amongst our colleagues for their input. Based on these discussions we have now modified this figure and moved it to **Supp Fig 2D** (in the updated version). We darkened the lines that set up the various response

quadrants. We changed the wording “IFN-independent” to “with or without IFN”. However, we decided to keep the terms "antiviral" and proviral". We created a new **Fig 2** (in the updated version), that shows some pathways of interest in a more intuitive fashion (showing the z-score for each gene).

6. Figure 4:

Comment: Are these data from a one experiment performed on one day with replicates in independent wells? Can you state in the figure legend if this is all from one experiment on one day? To say this is "independent" wouldn't it need to be done with different cells on different days? Or do you consider same cells different well infected with same virus batch "independent"?

Response: We have clarified in the figure legend that the data are from separate wells infected on the same day.

7. Figure 5:

Comment: Fig 5 probably better fits in supplemental data.

Response: We have moved **Fig 5** (in the previous version) to **Supp Fig 6** (in the updated version).

8. Supplementary Figure 5:

Comment: Can you put stats on A-I? Again are these independent infections all from one round of infection done on one day? If so is this a technical replicate vs. biological replicate? Please state in the figure legend.

Response: We have added stats to A-I and clarified the nature of the replicates in the figure legend in **Supp Fig 9** in the updated version (**Supp Fig 5** in the previous version).

9. Figure 7F-G:

Comment: This is was a bit confusing on first pass. Can you enhance your description of this experiment in the main text? Mention 7F is analysis of A-E in text (I see its in the legend but it confused me for a second so might be easier to have a couple words in the main text). For 7G, this is plaque assay rather than just # positive cells I think. Maybe include photos of these results as well? There is room in Fig 7 and its nice to see visually what the data looks like.

Response: We have included photos and rewritten the text related to **Fig 6** in the updated version (**Fig 7** in the previous version) for enhanced clarity. Please see the Results section:

During the COVID-19 pandemic, SARS-CoV-2 variants evolved from the initial strain, showing increased immune evasion and transmissibility [123-125]. To examine if these variants could circumvent the antiviral action of PLSCR1, we infected WT and PLSCR1 KO Huh-7.5 cells with an early strain isolated in July 2020 (NY-RU-NY1, subsequently referred to as "parental") and the Beta (B.1.352), Delta (B.1.617.2), Omicron (BA.5), and Omicron (XBB.1.5) variants. PLSCR1 continued to restrict these later variants when examining the percentage of infected cells 24 hours post-infection (Figs 6A-E).

To determine if the magnitude of PLSCR1 restriction was the same for the parental SARS-CoV-2 strain and recent variants, we plotted the percentage of infection data from Figs 6A-E as a ratio of PLSCR1 WT to KO (Fig 6F). Recent SARS-CoV-2 variants showed reduced differences in infection rates between PLSCR1 WT and KO cells than the parental SARS-CoV-2 strain. The diminished difference in sensitivity between PLSCR1 WT and KO cells was most pronounced with Omicron BA.5 and its descendant, XBB.1.5 (Fig 6F).

To examine this further, we infected PLSCR1 WT and KO Huh-7.5 cells with approximately 50 focus-forming units (FFU) per well for different SARS-CoV-2 strains (Figs 6G and 6H). In line with Fig 6F, the data indicate that the difference in virus susceptibility between PLSCR1 WT and KO cells is lower for more recent variants such as Beta (B.1.352) and especially Omicron (XBB.1.5) compared with the parental strain (Fig 6H).

Comment: For all of fig 6 and 7 clarify the KO is PLSCR1 in the various panels.

Response: We have clarified that the KO is PLSCR1 in the various panels of **Figs 5 and 6** in the updated manuscript (**Figs 6 and 7** in the previous version).

11. Figure 8A:

Comment: Label the gene PLSCR1 above the diagram to help with clarity and ease of rapid interpretation.

Response: We have labeled PLSCR1 in **Fig 7A** in the updated version (**Fig 8A** in the previous version).

Reviewer #2

General Comments

Comment: This manuscript identifies PLSCR1 as an effective IFN-independent antiviral protein. It is clearly written, and results are well presented. A very similar paper was published several months ago (Xu et al, Nature, 2023). Compared to Xu et al., additional or somewhat different results this manuscript shows include (1) PLSCR1-mediated restriction can be overcome by TMPRSS2 over-expression, (2) more recent SARS-CoV-2 variants are less restricted by PLSCR1 than the earlier strain, and (3) use of fibroblasts derived from patients carrying heterozygous H262Y mutation in PLSCR1. To gain more insights from these differences, a couple small experiments are suggested below. Together with the study by Xu et al. and the results from the suggested experiments, this study will provide insights into a novel anti-viral mechanism.

Response: Thank you for your positive feedback and for highlighting the clarity and quality of our manuscript. We appreciate your recognition of the additional insights our study provides compared to the excellent work from Xu et al. [2]. We would like to add that our study constitutes the first whole genome arrayed CRISPR KO screen in virus-infected cells and serves as a unique resource compiling a large number of published SARS-CoV-2 and COVID-19 studies.

Major Comments:

1. TMPRSS2 Dependency:

Comment: Because PLSCR1-mediated restriction can be overcome by TMPRSS2, to confirm that newer SARS-coV-2 variants are less restricted by PLSCR1 the authors should either show more recent SARS-coV-2 variant, XBB.1.5, is less dependent on TMPRSS2. If the result is different from expected, possible explanations should be given.

Response: Due to time constraints, we did not complete a side-by-side comparison of entry pathways between different SARS-CoV-2 strains. However, we cite studies in the Discussion section that have conducted such comparisons [3-5]. XBB.1.5 can overcome PLSCR1-

mediated restriction in cells lacking TMPRSS2, indicating that there must be other mechanisms for escaping PLSCR1 besides TMPRSS2-dependent entry. To clarify this, we added the following to the Results section:

Our data suggest that PLSCR1 restricts newer SARS-CoV-2 variants less efficiently in Huh-7.5 cells. This could be due to adaptation of the recent variants to directly antagonize PLSCR1 and/or utilize an alternative entry route in Huh-7.5 cells that is TMPRSS2-independent and invulnerable to PLSCR1.

We added in the Discussion:

We postulate that Omicron evolved its entry pathway to evade PLSCR1-mediated restriction while shifting its cell tropism to the upper airways and away from TMPRSS2-expressing cells [163, 165-169].

Future research should investigate: (i) mechanism(s) by which Omicron evades PLSCR1 restriction and whether this evasion is primarily due to mutations in the spike protein, (ii) the association between PLSCR1-mediated restriction and the distinct entry mechanisms utilized by SARS-CoV-2 variants, (iii) how various intrinsic factors that restrict virus entry, such as PLSCR1, have influenced SARS-CoV-2 entry routes, evolution, and cell tropism. Understanding these aspects of PLSCR1 restriction could provide mechanistic insight into broad strategies of PLSCR1 evasion employed by both newer SARS-CoV-2 variants as well as viruses from other families that were unaffected by PLSCR1 KO. Subversion of PLSCR1 restriction may serve as a useful immune evasion strategy that could be shared by diverse viruses with longer evolutionary selection than SARS-CoV-2.

---

2. TMPRSS2 Expression in Huh7.5:

Comment: The authors stated SARS-CoV-2 infection of Huh7.5 cells does not depend on TMPRSS2, because its KO did not influence infection (lines 152-155). If Huh7.5 expresses

TMPRSS2, show its expression before and after its KO. If Huh7.5 does not express TMPRSS2, it should be stated in the text.

Response: Indeed, Huh-7.5 cells do not express TMPRSS2. We now show a TMPRSS2 western blot (**Supp Fig 1B** in the updated manuscript) and discuss it in the text, see Results section:

Similar to A549-ACE2 cells [11, 36], Huh-7.5 cells do not express transmembrane serine protease 2 (TMPRSS2) (Supp Fig 1B). As a result, SARS-CoV-2 entry is restricted to the endocytic route [37, 38].

---

3. H262Y Mutation:

Comment: As H262Y mutation is present in the region for nuclear localization signal, does this mutation affect nuclear localization of PLSCR1? If yes, the authors should discuss why restriction by PLSCR1 of beta coronaviruses whose genome does not require nuclear transport is affected by the H262Y mutation. If H262Y mutation does not affect nuclear localization of PLSCR1, describe so.

Response: We thank the reviewer for bringing up this interesting point. We now show that H262Y does not alter the fact that PLSCR1 is primarily localized in the cytoplasm (**Supp Fig 12**) and discuss it in the text, see Results section:

Since the p.His262Tyr mutation is located in the NLS region, we hypothesized that it could impair PLSCR1's nuclear localization. However, PLSCR1 WT was primarily localized in the cytoplasm in untreated A549-ACE2 cells (Fig 5A), in IFN-treated cells (Supp Fig 11B), and in SARS-CoV-2 infected cells (Supp Fig 11C). Similarly, PLSCR1 His262Tyr was also enriched in the cytoplasm (Supp Fig 12). This suggests that the palmitoylation motif, known to be dominant over the NLS [129], dictates the cytoplasmic localization of PLSCR1. Therefore, we propose that the p.His262Tyr mutation affects PLSCR1 through a mechanism other than altering its nuclear localization.

Minor Comments:

1. Assertion on intrinsic PLSCR1:

Comment: Line 224: "intrinsic PLSCR1 contributes to the restriction of SARS-CoV-2, even without IFN. As we do not know if this is true in vivo, the statement should be limited to 'in vitro' or 'in cell lines'.

Response: We have added single-cell RNA-seq data from [6], showing that PLSCR1 is constitutively expressed in SARS-CoV-2 target cells in vivo (**Supp Fig 5D**) and discussed this in the text. Additionally, we clarified that our functional data pertains to cell culture. Please see the Results section for these updates:

Overall, our data indicate that intrinsic PLSCR1 restricts SARS-CoV-2 in cell culture, even in the absence of IFN. Given that PLSCR1 mRNA is constitutively expressed in SARS-CoV-2 target cells (Supp Fig 5D) [118], its intrinsic antiviral function may also be effective in vivo.

---

2. Biosafety Level:

Comment: Replication-competent CHIKV and VEEV were used in the study. That they were handled only in the biosafety level 3 should be indicated.

Response: These are BSL-2 vaccine strains of CHIKV and VEEV and were handled using biosafety level 2 containment and practices. All virus strains are referenced in the Methods section.

3. Patient-Derived Fibroblasts:

Comment: Patient-derived fibroblasts carrying a heterozygous mutant (H262Y) in PLSCR1 were used in the study. The source of these cells should be given. Unless they are commercial, IRB should be in order.

Response: Thank you for pointing out this omission. We have now included the following in the Methods section:

Written informed consent was obtained in the country of residence of the patients, in accordance with local regulations, and with institutional review board (IRB) approval. Experiments were conducted in the United States in accordance with local regulations and with the approval of the IRB.

Approval was obtained from the Rockefeller University Institutional Review Board in New York, USA (protocol no. JCA-0700).

Comment: Are these COVID-19 patients? If not, disease name should be given. If they are COVID-19 patients, more information (the year and month cells were obtained, disease status, how long hospitalized, and etc) should be given or cite the source for such information. It may help and strengthen the authors' claim.

Response: These are not COVID-19 patients. We clarified this point in the text, see Results section:

To counter this, we examined patient-derived SV40-immortalized fibroblasts expressing ACE2 that were heterozygous for p.His262Tyr. These cells were already present in our collection, originating from a female tuberculosis patient from Turkey (JL0192) and a female herpes simplex encephalitis patient from France (JL0480). The origin of these cells is serendipitous; our goal was not to link these diseases to PLSCR1 but rather to infect cells carrying the His262Tyr variant with SARS-CoV-2.

---

5. Immune Evasion:

Comment: Lines 354-5 say "...evolved to bind PLSCR1 as a mechanism of immune evasion." The authors may want to elaborate on how binding to PLSCR1 helps viruses to evade immune mechanisms..

Response: We do not currently know what these mechanisms might be. We added in the Discussion:

An alternative explanation is that diverse viral proteins convergently evolved to bind PLSCR1 as a mechanism of immune evasion. For example, by altering PLSCR1's subcellular localization.

Reviewer #3

General comments

Comment: The manuscript by Le Pen et al presents the results of an arrayed CRISPR screen to identify host factors that are either required for, or restrict, SARS-CoV-2 replication +/- IFN treatment. Amongst other factors the authors identify is PLSCR1, a membrane protein of unknown function. PLSCR1 expression restricts viral entry and is eliminated abolished by TMPRSS2 overexpression and appears to be reduced for later SARS-CoV-2 VoCs. Lastly a coding polymorphism in PSCLR1 has been associated with SARS-CoV-2 susceptibility. The authors show that cells from individuals with this polymorphism are more susceptible to infection and ectopic expression of this mutant has an attenuated restriction activity.

This is a well performed study and the results are of interest. The comparison with other screens is welcome. The downside is of course that a recent study has already identified that PSCLR1 is able to block the entry of SARS CoV-2. While the authors have added some further data (the human polymorph and some other viruses) the mechanistic understanding of PSCLR1 function is quite underdeveloped at present and in the light of previous publications more insight here would add value to this paper over and above a confirmatory study. The authors should address some the following:

Response: We appreciate the reviewer's positive feedback on our study and their recognition of its interest and value. We acknowledge that a recent study [2] has identified PLSCR1 as a factor that blocks SARS-CoV-2 entry, and we agree that further mechanistic insights into PLSCR1 function would be of great interest to the community. Given that our submission falls under PLOS Biology's anti-scooping policy and the urgency of publication, we could not address all the requests from the reviewer. However, we have addressed several key points to the best of our ability.

Major Comments

1. Viral Growth Curves:

Comment: I'm not particularly persuaded by the current data of the resistance of later VOCs to PSCLR1 without some more robust viral growth curves.

Response: We have added viral growth curves that support our conclusions in **Figs 6I and 6J** in the updated version.

Comment: Given that spike is sufficient to confer PSCLR1 sensitivity to HIV vectors, it should be straightforward to show that relative resistance is spike determined and if so to map the determinant in spike responsible. Since restriction is operating at entry and that TMPRSS2 expression reduces the sensitivity to PSCLR1, an obvious place to look would be the polybasic cleavage site and the adaptations associated with it.

Response: We attempted to model the PLSCR1 sensitivity using HIV vectors pseudotyped with WT spike (as in **Figs 5F** and **5L** in the updated version) as well as the spike protein from recent SARS-CoV-2 variants. We started by generating a bulk population of HEK293T-ACE2 PLSCR1 KO cells because HEK293T-ACE2 are more permissive to spike-pseudotyped HIV than Huh-7.5 or A549-ACE2 cells and thus better suited for this study. Then, we transduced these cells with lentiviruses pseudotyped with the envelope proteins of interest and measured entry using an EGFP reporter carried by the lentiviruses. The attached data (**Figs below**) support our conclusions and replicate the findings with WT spike-pseudotyped lentiviruses shown in **Fig 5L** (in the updated version). Note that **Fig 5L** uses siRNA-mediated KD for PLSCR1 LOF versus CRISPR KO in the **Figs below**. However, the effect of PLSCR1 on the WT spike-mediated entry is not as pronounced with the pseudotyped lentiviruses than with the live SARS-CoV-2 viruses. This could notably be because KO is incomplete in the bulk HEK293T-ACE2 PLSCR1 KO cells shown in the **Figs below** or because other SARS-CoV-2 proteins than spike influence PLSCR1 sensitivity. Ultimately, this question, although of great interest, would demand a new set of tools, clonal cell lines, and orthogonal models for spike-mediated virus-cell membrane fusion. This is an interesting area for future study, that we believe is beyond the scope of this current investigation.

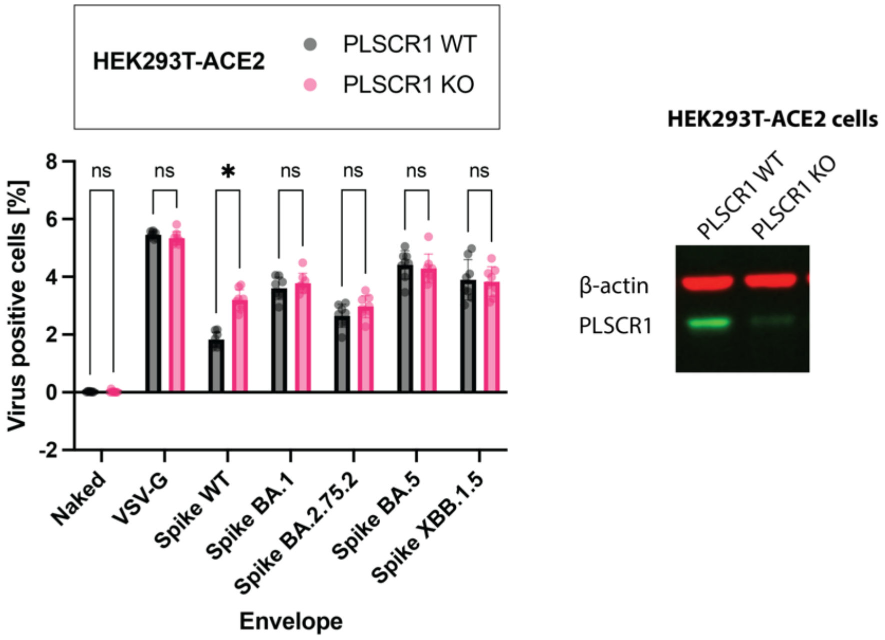

Left panel: HEK293T-ACE2 cells, PLSCR1 WT or PLSCR1 KO (bulk population) transduction with HIV-based replication defective virus with neon green reporter, pseudotyped with envelope proteins as indicated. n = 8 separate wells transduced on the same day. Error bars = sd. ns, non-significant; *, $p \leq 0.05$; multiple unpaired t tests. **Right panel**: PLSCR1 western blot on the cells used in left panel.

2. NCOA7 and IFITMs:

Comment: NCOA7 and IFITMs differentially affect SARS-CoV-2 entry dependent on S and proteases. Is PSCLR1 restriction associated with their activities?

Response: This is an interesting point. We now include data on the genetic association between *PSCLR1* and some other ISGs blocking SARS-CoV-2 entry, see Results section:

Other ISG products have been described to restrict SARS-CoV-2 entry (Supp Fig 1) [1], notably: CH25H promotes cholesterol sequestration in lipid droplets, decreasing the pool of accessible cholesterol required for virus-cell membrane fusion [28,29]; LY6E blocks virus-cell membrane fusion via currently undetermined mechanisms [121]; nuclear receptor coactivator 7 (NCOA7) over-acidifies the lysosome, leading to viral antigen degradation by lysosomal proteases [31,32]; and interferon induced transmembrane protein 2 (IFITM2) blocks pH- and cathepsin-dependent SARS-CoV-2 virus-cell membrane fusion in the endosome [122]. The aforementioned ISGs were not hits in our CRISPR KO screen for antiviral genes in IFN-treated cells. This may be due to functional redundancies among them. We found

that over-expression of any of the aforementioned ISGs restricted SARS-CoV-2 in PLSCR1 KO cells, indicating that they do not need PLSCR1 for their antiviral activity (Supp Fig 8A). PLSCR1 is unlikely to require CH25H, LY6E, or IFITM2 for its function as these are expressed at minimal levels in Huh-7.5 cells without IFN pre-treatment (Supp Fig 8B,C). We cannot exclude an association between PLSCR1 and NCOA7, although the latter was not hit in our screen.

We have also added to the Discussion section:

Several PLSCR1 variants were enriched in a GWAS on severe COVID-19, with a relatively low odds ratio of approximately 1.2 [25, 26]. This modest odds ratio likely reflects the complex redundancies within antiviral defenses, from innate immunity featuring multiple effector ISGs that restrict SARS-CoV-2 [10-15, 18, 19], to adaptive immunity [170]. For example, we show that the ISGs CH25H [28,29], IFITM2 [122], LY6E [121], and NCOA7 [31,32] still function to restrict SARS-CoV-2 in PLSCR1-depleted cells (Supp Fig 8).

---

3. Human Polymorph Insight:

Comment: Is there any obvious insight into why the human polymorph is less restrictive? The H262Y change maps to an apparent NLS (although whether this is real given the protein in membrane anchored is unclear). While similarly expressed, does this protein localize like the wildtype?

Response: Please see the response to reviewer 2 Major Comments #3.

4. ACE2 Surface Levels:

Comment: How does PSCLR1 expression affect ACE2 surface levels?

Response: We have included data showing that PSCLR1 expression does not significantly affect ACE2 surface levels, see Results section:

One hypothesis for PLSCR1's specificity for SARS-CoV-2 is that it alters the surface levels of its receptor, ACE2. However, flow cytometry on live cells did not show a significant effect of PLSCR1 on ACE2 surface levels in A549-ACE2 cells (Supp Fig 10). The precise mechanism of action of PLSCR1 remains undetermined.

---

5. PSCLR1 Specificity:

Comment: The specificity of PSCLR1 restriction to SARS-CoV-2 and no other enveloped virus tested including other seasonal CoVs is striking. Especially as some others appear to require it for replication. How widespread is PSCLR1 restriction amongst other sarbecovirus spikes?

Response: This is an interesting area for future study, that we believe is beyond the scope of this current investigation.

Minor Comments

1. IFITM Proteins:

Comment: IFITM proteins are missing from figure 1.

Response: We thank the reviewer for pointing out this omission. We have updated **Supp Fig 1A** in the updated version (**Figure 1** in the previous version) to include the IFITM proteins. The figure legend reads:

The role of IFITM1-3 proteins in SARS-CoV-2 entry has been the subject of numerous studies, which have sometimes yielded varying conclusions [1, 122, 199-205]. However, it appears that (i) IFITM2 restricts WT SARS-CoV-2 endosomal entry [122], (ii) recent SARS-CoV-2 variants may evade IFITM2 [199], and (iii) IFITM3 KO mice are hyper-susceptible to WT SARS-CoV-2 [206].

---

2. Terminology:

Comment: While "kraken" was a name that got traction on social media to describe XBB 1.5, it is part of the omicron grouping and probably shouldn't be used in scientific papers.

Response: We have replaced "kraken" with "Omicron (XBB 1.5)" in the manuscript.

We hope these revisions address the reviewers' comments and improve the clarity and impact of our manuscript. We thank the reviewers for their valuable feedback and look forward to your favorable consideration.

Sincerely,

Jérémie Le Pen

References

1. Cross BC, Lawo S, Archer CR, Hunt JR, Yarker JL, Riccombeni A, Little AS, McCarthy NJ, Moore JD: **Increasing the performance of pooled CRISPR-Cas9 drop-out screening**. *Sci Rep* 2016, **6**:31782.
2. Xu D, Jiang W, Wu L, Gaudet RG, Park ES, Su M, Cheppali SK, Cheemarla NR, Kumar P, Uchil PD, et al.: **PLSCR1 is a cell-autonomous defence factor against SARS-CoV-2 infection**. *Nature* 2023, **619**:819-827.
3. Willett BJ, Grove J, MacLean OA, Wilkie C, De Lorenzo G, Furnon W, Cantoni D, Scott S, Logan N, Ashraf S, et al.: **SARS-CoV-2 Omicron is an immune escape variant with an altered cell entry pathway**. *Nat Microbiol* 2022, **7**:1161-1179.
4. Peacock TP, Brown JC, Zhou J, Thakur N, Sukhova K, Newman J, Kugathasan R, Yan AWC, Furnon W, Lorenzo GD, et al.: **The altered entry pathway and antigenic distance of the SARS-CoV-2 Omicron variant map to separate domains of spike protein**. *bioRxiv* 2022:2021.2012.2031.474653.
5. Shi G, Li T, Lai KK, Johnson RF, Yewdell JW, Compton AA: **Omicron Spike confers enhanced infectivity and interferon resistance to SARS-CoV-2 in human nasal tissue**. *Nat Commun* 2024, **15**:889.
6. Abdulla S, Aevermann B, Assis P, Badajoz S, Bell SM, Bezzi E, Cakir B, Chaffer J, Chambers S, Michael Cherry J, et al.: **CZ CELL×GENE Discover: A single-cell data platform for scalable exploration, analysis and modeling of aggregated data**. *bioRxiv* 2023:2023.2010.2030.563174.
