# Supplementary material for: A genome-wide arrayed CRISPR screen identifies PLSCR1 as an intrinsic barrier to SARS-CoV-2 entry that recent virus variants have evolved to resist
Source: PLoS Biol. 2024 Sep 24;22(9):e3002767. doi: 10.1371/journal.pbio.3002767 (PMC11486371; doi:10.1371/journal.pbio.3002767)
Supplement: Attachment — Submitted filename: Point by point response to editor.docx [file pbio.3002767.s039.docx]

Dear Dr Le Pen,


a) We would like to suggest the following modification to the title:

"A genome-wide arrayed CRISPR screen identifies PLSCR1 as an intrinsic barrier to SARS-CoV-2 entry that recent virus variants have evolved to resist"

Thank you for the suggestion. We modified the title.

b) You may be aware of the PLOS Data Policy, which requires that all data be made available without restriction: [https://urldefense.com/v3/__http://journals.plos.org/plosbiology/s/data-availability__;!!M59pwtRysTUf!YN7fRU39q3wkK8sGtRbuKVovMciVvOzQHpZsarSfCoqb1uM2aUQ8qLJ0VK0rNjuXC38eKVkbnKXtr98Bnl3o5Q$](https://urldefense.com/v3/__http:/journals.plos.org/plosbiology/s/data-availability__;!!M59pwtRysTUf!YN7fRU39q3wkK8sGtRbuKVovMciVvOzQHpZsarSfCoqb1uM2aUQ8qLJ0VK0rNjuXC38eKVkbnKXtr98Bnl3o5Q$) . For more information, please also see this editorial: [https://urldefense.com/v3/__http://dx.doi.org/10.1371/journal.pbio.1001797__;!!M59pwtRysTUf!YN7fRU39q3wkK8sGtRbuKVovMciVvOzQHpZsarSfCoqb1uM2aUQ8qLJ0VK0rNjuXC38eKVkbnKXtr9-STcqqMQ$](https://urldefense.com/v3/__http:/dx.doi.org/10.1371/journal.pbio.1001797__;!!M59pwtRysTUf!YN7fRU39q3wkK8sGtRbuKVovMciVvOzQHpZsarSfCoqb1uM2aUQ8qLJ0VK0rNjuXC38eKVkbnKXtr9-STcqqMQ$)

Please supply the numerical values either in the a supplementary file or as a permanent DOI’d deposition for the following figures:
Figure 1ABCEF, 2, 4ABC, 5CDEFGHIJKLM, 6ABCDEFHIJ, 7BCDE, S2ABC, S5BD, S6ABCDEFGHIJ, S7BC, S8ABC, S9ABCDEFGHIJKLM, S14
Done, see Supp Table 1.

NOTE: the numerical data provided should include all replicates AND the way in which the plotted mean and errors were derived (it should not present only the mean/average values).

c) Please cite the location of the data clearly in all relevant main and supplementary Figure legends, e.g. “The data underlying this Figure can be found in S1 Data” or “The data underlying this Figure can be found in [https://urldefense.com/v3/__https://doi.org/10.5281/zenodo.XXXXX__;!!M59pwtRysTUf!YN7fRU39q3wkK8sGtRbuKVovMciVvOzQHpZsarSfCoqb1uM2aUQ8qLJ0VK0rNjuXC38eKVkbnKXtr9_jGI8ibw$](https://urldefense.com/v3/__https:/doi.org/10.5281/zenodo.XXXXX__;!!M59pwtRysTUf!YN7fRU39q3wkK8sGtRbuKVovMciVvOzQHpZsarSfCoqb1uM2aUQ8qLJ0VK0rNjuXC38eKVkbnKXtr9_jGI8ibw$) ”
Done, see Supp Table 1.

d) We require the original, uncropped and minimally adjusted images supporting all blot and gel results reported in the Figures:
Figure S1B, S5AC, S7A, S11
Now available on Dryad (DOI: 10.5061/dryad.6q573n65k), see Supp Information 1.

We will require these files before a manuscript can be accepted so please prepare and upload them now. Please carefully read our guidelines for how to prepare and upload this data: [https://urldefense.com/v3/__https://journals.plos.org/plosbiology/s/figures*loc-blot-and-gel-reporting-requirements__;Iw!!M59pwtRysTUf!YN7fRU39q3wkK8sGtRbuKVovMciVvOzQHpZsarSfCoqb1uM2aUQ8qLJ0VK0rNjuXC38eKVkbnKXtr98VTWJ_ug$](https://urldefense.com/v3/__https:/journals.plos.org/plosbiology/s/figures*loc-blot-and-gel-reporting-requirements__;Iw!!M59pwtRysTUf!YN7fRU39q3wkK8sGtRbuKVovMciVvOzQHpZsarSfCoqb1uM2aUQ8qLJ0VK0rNjuXC38eKVkbnKXtr98VTWJ_ug$)

e) “For figures containing FACS data (Figure S10), please provide the FCS files and a picture showing the successive plots and gates that were applied to the FCS files to generate the figure. We ask that you please deposit this data in the FlowRepository ([https://urldefense.com/v3/__https://flowrepository.org/__;!!M59pwtRysTUf!YN7fRU39q3wkK8sGtRbuKVovMciVvOzQHpZsarSfCoqb1uM2aUQ8qLJ0VK0rNjuXC38eKVkbnKXtr9_9rtAeZQ$](https://urldefense.com/v3/__https:/flowrepository.org/__;!!M59pwtRysTUf!YN7fRU39q3wkK8sGtRbuKVovMciVvOzQHpZsarSfCoqb1uM2aUQ8qLJ0VK0rNjuXC38eKVkbnKXtr9_9rtAeZQ$) ) and provide the accession number/URL of the deposition in the Data Availability Statement in the online submission form.”
Now available on Dryad (DOI: 10.5061/dryad.6q573n65k), see Supp Information 2 and 3.

e) Please ensure that your Data Statement in the submission system accurately describes where your data can be found and is in final format, as it will be published as written there.

Done.

f) We thank you for uploading the supplemental data to Dryad. However, we were not able to access it and evaluate the data deposited there. Please make sure to allow access before submitting it again.

I just made a new submission and made it public. It should be released soon by the Dryad team. Here is the current info:

Your dataset has been assigned two important links:

- Reviewer URL: [https://datadryad.org/stash/share/rcZlA4tALKUjVkOioaNvnD1uOAXMz1PTHyLCJTVNWcY](https://urldefense.com/v3/__https:/datadryad.org/stash/share/rcZlA4tALKUjVkOioaNvnD1uOAXMz1PTHyLCJTVNWcY__;!!M59pwtRysTUf!ZR_kQk5U5IFAoMeVeCwjWcBo-qGI7xn3KdsK-ahHIqXUmZ-BKZsAW6DPXIBGN6V1iHBqFhWuSkkgwWefpdCPUDpS0qKePfk9Ww$).

This temporary link can be shared with the journal office or collaborators during the peer-review process to allow access only to the data files included with your submission. This link will expire upon publication of your Dryad dataset.

- A unique digital object identifier (DOI): doi:10.5061/dryad.6q573n65k

When your dataset has been approved for publication by a member of our curation team, it will be made public. This DOI link is permanent and should be provided to the journal office for inclusion in the Data Availability statement of your published article.

f) Per journal policy, if you have generated any custom code during the curse of this investigation, please make it available without restrictions upon publication. Please ensure that the code is sufficiently well documented and reusable, and that your Data Statement in the Editorial Manager submission system accurately describes where your code can be found.

Please note that we cannot accept sole deposition of code in GitHub, as this could be changed after publication. However, you can archive this version of your publicly available GitHub code to Zenodo. Once you do this, it will generate a DOI number, which you will need to provide in the Data Accessibility Statement (you are welcome to also provide the GitHub access information). See the process for doing this here: [https://urldefense.com/v3/__https://docs.github.com/en/repositories/archiving-a-github-repository/referencing-and-citing-content__;!!M59pwtRysTUf!YN7fRU39q3wkK8sGtRbuKVovMciVvOzQHpZsarSfCoqb1uM2aUQ8qLJ0VK0rNjuXC38eKVkbnKXtr98JLVH52Q$](https://urldefense.com/v3/__https:/docs.github.com/en/repositories/archiving-a-github-repository/referencing-and-citing-content__;!!M59pwtRysTUf!YN7fRU39q3wkK8sGtRbuKVovMciVvOzQHpZsarSfCoqb1uM2aUQ8qLJ0VK0rNjuXC38eKVkbnKXtr98JLVH52Q$)

Now available on Dryad (DOI: 10.5061/dryad.6q573n65k).

As you address these items, please take this last chance to review your reference list to ensure that it is complete and correct. If you have cited papers that have been retracted, please include the rationale for doing so in the manuscript text, or remove these references and replace them with relevant current references. Any changes to the reference list should be mentioned in the cover letter that accompanies your revised manuscript.

The Rebendenne et al. citation has been updated from the preprint to the peer-reviewed journal.

Many thanks.

With best wishes,

Jérémie


We expect to receive your revised manuscript within two weeks. 

To submit your revision, please go to [https://urldefense.com/v3/__https://www.editorialmanager.com/pbiology/__;!!M59pwtRysTUf!YN7fRU39q3wkK8sGtRbuKVovMciVvOzQHpZsarSfCoqb1uM2aUQ8qLJ0VK0rNjuXC38eKVkbnKXtr9_tR6X5Gw$](https://urldefense.com/v3/__https:/www.editorialmanager.com/pbiology/__;!!M59pwtRysTUf!YN7fRU39q3wkK8sGtRbuKVovMciVvOzQHpZsarSfCoqb1uM2aUQ8qLJ0VK0rNjuXC38eKVkbnKXtr9_tR6X5Gw$)  and log in as an Author. Click the link labelled 'Submissions Needing Revision' to find your submission record. Your revised submission must include the following:
-  a cover letter that should detail your responses to any editorial requests, if applicable, and whether changes have been made to the reference list
-  a Response to Reviewers file that provides a detailed response to the reviewers' comments (if applicable, if not applicable please do not delete your existing 'Response to Reviewers' file.)
-  a track-changes file indicating any changes that you have made to the manuscript. 

NOTE: If Supporting Information files are included with your article, note that these are not copyedited and will be published as they are submitted. Please ensure that these files are legible and of high quality (at least 300 dpi) in an easily accessible file format. For this reason, please be aware that any references listed in an SI file will not be indexed. For more information, see our Supporting Information guidelines:
[https://urldefense.com/v3/__https://journals.plos.org/plosbiology/s/supporting-information__;!!M59pwtRysTUf!YN7fRU39q3wkK8sGtRbuKVovMciVvOzQHpZsarSfCoqb1uM2aUQ8qLJ0VK0rNjuXC38eKVkbnKXtr98ovcythA$](https://urldefense.com/v3/__https:/journals.plos.org/plosbiology/s/supporting-information__;!!M59pwtRysTUf!YN7fRU39q3wkK8sGtRbuKVovMciVvOzQHpZsarSfCoqb1uM2aUQ8qLJ0VK0rNjuXC38eKVkbnKXtr98ovcythA$)   

*Published Peer Review History*
Please note that you may have the opportunity to make the peer review history publicly available. The record will include editor decision letters (with reviews) and your responses to reviewer comments. If eligible, we will contact you to opt in or out. Please see here for more details:
[https://urldefense.com/v3/__https://blogs.plos.org/plos/2019/05/plos-journals-now-open-for-published-peer-review/__;!!M59pwtRysTUf!YN7fRU39q3wkK8sGtRbuKVovMciVvOzQHpZsarSfCoqb1uM2aUQ8qLJ0VK0rNjuXC38eKVkbnKXtr9-ubsC4QQ$](https://urldefense.com/v3/__https:/blogs.plos.org/plos/2019/05/plos-journals-now-open-for-published-peer-review/__;!!M59pwtRysTUf!YN7fRU39q3wkK8sGtRbuKVovMciVvOzQHpZsarSfCoqb1uM2aUQ8qLJ0VK0rNjuXC38eKVkbnKXtr9-ubsC4QQ$)

*Press*
Should you, your institution's press office or the journal office choose to press release your paper, please ensure you have opted out of Early Article Posting on the submission form. We ask that you notify us as soon as possible if you or your institution is planning to press release the article.

*Protocols deposition*
To enhance the reproducibility of your results, we recommend that if applicable you deposit your laboratory protocols in [protocols.io](http://protocols.io), where a protocol can be assigned its own identifier (DOI) such that it can be cited independently in the future. Additionally, PLOS ONE offers an option for publishing peer-reviewed Lab Protocol articles, which describe protocols hosted on [protocols.io](http://protocols.io). Read more information on sharing protocols at [https://urldefense.com/v3/__https://plos.org/protocols?utm_medium=editorial-email&utm_source=authorletters&utm_campaign=protocols__;!!M59pwtRysTUf!YN7fRU39q3wkK8sGtRbuKVovMciVvOzQHpZsarSfCoqb1uM2aUQ8qLJ0VK0rNjuXC38eKVkbnKXtr98SLX3Feg$](https://urldefense.com/v3/__https:/plos.org/protocols?utm_medium=editorial-email&utm_source=authorletters&utm_campaign=protocols__;!!M59pwtRysTUf!YN7fRU39q3wkK8sGtRbuKVovMciVvOzQHpZsarSfCoqb1uM2aUQ8qLJ0VK0rNjuXC38eKVkbnKXtr98SLX3Feg$)


Please do not hesitate to contact me should you have any questions.

Sincerely,

Melissa
